# Category-Based Toxicokinetic Evaluations of Data-Poor Per- and Polyfluoroalkyl Substances (PFAS) using Gas Chromatography Coupled with Mass Spectrometry

**DOI:** 10.3390/toxics11050463

**Published:** 2023-05-16

**Authors:** Anna Kreutz, Matthew S. Clifton, W. Matthew Henderson, Marci G. Smeltz, Matthew Phillips, John F. Wambaugh, Barbara A. Wetmore

**Affiliations:** 1 Oak Ridge Institute for Science and Education, 1299 Bethel Valley Road, Oak Ridge, TN 37830, USA; anna.kreutz@nih.gov; 2 US Environmental Protection Agency, Office of Research and Development, Center for Environmental Measurement and Modeling, Research Triangle Park, NC 27711, USA; clifton.matthew@epa.gov (M.S.C.); smeltz.marci@epa.gov (M.G.S.); 3 US Environmental Protection Agency, Office of Research and Development, Center for Environmental Measurement and Modeling, Athens, GA 30605, USA; henderson.matt@epa.gov; 4 US Environmental Protection Agency, Office of Research and Development, Center for Computational Toxicology and Exposure, Research Triangle Park, NC 27711, USA; wambaugh.john@epa.gov; 5 Oak Ridge Associated Universities, 100 ORAU Way, Oak Ridge, TN 37830, USA

**Keywords:** PFAS, toxicokinetics, in vitro–in vivo extrapolation, plasma protein binding, hepatic clearance, new approach methods, biotransformation

## Abstract

Concern over per- and polyfluoroalkyl substances (PFAS) has increased as more is learned about their environmental presence, persistence, and bioaccumulative potential. The limited monitoring, toxicokinetic (TK), and toxicologic data available are inadequate to inform risk across this diverse domain. Here, 73 PFAS were selected for in vitro TK evaluation to expand knowledge across lesser-studied PFAS alcohols, amides, and acrylates. Targeted methods developed using gas chromatography–tandem mass spectrometry (GC-MS/MS) were used to measure human plasma protein binding and hepatocyte clearance. Forty-three PFAS were successfully evaluated in plasma, with fraction unbound (f_up_) values ranging from 0.004 to 1. With a median f_up_ of 0.09 (i.e., 91% bound), these PFAS are highly bound but exhibit 10-fold lower binding than legacy perfluoroalkyl acids recently evaluated. Thirty PFAS evaluated in the hepatocyte clearance assay showed abiotic loss, with many exceeding 60% loss within 60 min. Metabolic clearance was noted for 11 of the 13 that were successfully evaluated, with rates up to 49.9 μL/(min × million cells). The chemical transformation simulator revealed potential (bio)transformation products to consider. This effort provides critical information to evaluate PFAS for which volatility, metabolism, and other routes of transformation are likely to modulate their environmental fates.

## 1. Introduction

Per- and polyfluoroalkyl substances (PFAS) comprise a class of anthropogenic compounds with diversity across molecular structures and chemical, physical, and biological properties [1,2]. The Organisation for Economic Co-operation and Development (OECD) defines PFAS as fluorinated substances that contain at least one fully fluorinated methyl or methylene carbon atom [2]. The OECD has assembled a list of 4730 substances meeting this criterion, ranging from volatile hydrofluorocarbons and perfluoroalkyl alkenes to the thermostable, longer carbon–fluorine chain-length carboxylic acids that are well known for their persistence, bioaccumulation, and health effects [3]. Although the chemical industry is transitioning away from reliance on longer chain-length PFAS, knowledge of alternative PFAS, either in development or now in use, is limited [4,5,6]. Consequently, regulatory agencies worldwide are faced with an imminent need for an actionable strategy to efficiently identify PFAS that are likely to require greater scrutiny for potential health and environmental hazards.

The 2021 release of the United States Environmental Protection Agency (US EPA) PFAS Strategic Roadmap outlines the use of in vitro toxicity testing and toxicokinetic (TK) studies to identify in vitro points of departure to broaden knowledge of PFAS bioactivity across multiple functional classes [7]. The goal is to identify what potencies or effects may be present for specific PFAS or categories of PFAS and to identify those warranting further investigation. Indeed, despite the phase-out of long-chain PFAS from production in the early 2000s, an inordinate amount of research continues to focus on these carboxylate and sulfonate moieties [3,8]. With an established framework in place for in vitro screening and decision making [9], new approach methods (NAMs) are positioned to address this need.

As with other xenobiotics, PFAS functional group presence conveys differing properties that may in turn elucidate new applications for PFAS in manufacturing and commerce. Fluorotelomers (FTs) such as FT alcohols (FTOHs) and FT iodides (FTIs) are employed in the telomerization manufacturing process, serving as precursors and intermediates in reactions to produce short-chain carboxylic acids [1]. Despite classification as a perfluoroalkyl acid (PFAA) precursor [1,10], FTOHs have also been employed in commercial products, with described uses and/or detections in various types of food packaging, cosmetics, cleaning products, impregnating sprays, and antifogging sprays and wipes (Table 1) [11,12,13,14,15,16,17]. Similarly, PFAS acrylates are used in the manufacturing of polymers as well as in personal care products and industrial uses, such as semiconductor manufacturing (Table 1). Even less is known in the public domain about per- and polyfluoroalkyl alcohols, perfluorinated alkanes, and per- and polyfluorinated amides, which meet the definition of PFAS but have not been commonly regarded as such [10]. Although recent scoping studies are beginning to elucidate uses [11,17], and nontargeted analyses are discovering many in consumer products [13,18], very little research is available on many of these groups to sufficiently evaluate environmental fates or human or environmental health risks.

Functional group presence will also modulate the environmental fate, transport, (bio)transformation, degradation, and persistence of each PFAS [19]. PFAA precursors such as FTOHs are well known for their conversion to perfluoroalkyl carboxylic acids (PFCAs), which can occur not just during intentional PFCA manufacturing, but also following microbial transformations in the environment or after mammalian exposure during in vivo studies [19,20].

FT acrylates are also used in the production of acrylate-linked fluorotelomer polymers (FTPs). Polymeric degradation as well as residual impurities in these polymers can be considered as environmental sources of these classes of compounds [21]. Volatility will also be a factor to consider in developing and testing exposure scenarios. For optimal detection and quantitation, these groups require the use of gas chromatography combined with tandem mass spectrometry and, in some instances, head-space analysis—approaches that are much less widely used than liquid chromatography–mass spectrometry. This is likely another factor contributing to the paucity of data on these PFAS groups.

NAMs provide numerous advantages over traditional in vivo toxicity studies—including eliminating the need for interspecies extrapolation, reducing costs, and allowing for examination of a range of endpoints that can be used in determining chemical modes of action. However, additional consideration of in vivo TK processes is required to incorporate dosimetry and estimate the administered equivalent dosages (AEDs) required to achieve internal concentrations consistent with in vitro measures of bioactivity [22]. Two of the most important TK parameters to consider are protein binding and hepatic clearance. The degree of protein binding determines the amount free to elicit an effect, be subject to metabolism, or to partition into cells or tissues. Hepatic clearance helps to determine the temporal profile of compound distribution and concentration [23]. Moreover, consideration of these parameters can also inform bioaccumulative potential, an important factor in evaluations of emerging contaminants such as PFAS [24,25].

In this effort, we have successfully developed targeted methods for 61 PFAS using gas chromatography–tandem mass spectrometry (GC-MS/MS) and/or GC-MS, with positive and negative chemical ionization (PCI and NCI, respectively). The original list of PFAS amenable to GC-MS/MS comprised 73 PFAS that spanned multiple functional groups and chemistries (Table 1). Human plasma protein binding (PPB) measures and hepatic clearance (Cl_int_) values were derived, and a Bayesian model was applied to capture experimental uncertainty, to inform PFAS bioaccumulative potential, metabolism, and dosimetry estimations. Abiotic stability in vitro was evaluated to characterize the potential for hydrolysis in aqueous environments. These findings offer key considerations, including analytical challenges, as scientists continue their attempts to characterize PFAS exposure and metabolic transformation, with the goal of developing suitable NAM testing strategies.

## 2. Materials and Methods

### 2.1. PFAS Stocks and Analytical Standards

PFAS were procured from a larger PFAS library through a US EPA contract with Evotec Inc. (Branford, CT). These are a subset of a larger group of over 140 PFAS that were selected to capture structural diversity and to facilitate read-across within categories, as described previously [26,27]. Those PFAS analyzed using liquid chromatography MS/MS have been described by Smeltz et al. and Crizer et al. [28,29]. Seventy-three PFAS of the overall set of one hundred forty were identified as being amenable to analysis using GC-MS/MS during the method-development phase. Table S1 lists the relevant PFAS along with vendor/purity, structural category, and physicochemical property information. Substances were solubilized in dimethyl sulfoxide (DMSO) at a target concentration of 30 mM, if possible, without precipitation. If visual inspection revealed that precipitation was an issue, they were solubilized at 10 mM. DMSO stocks of all selected PFAS passed an analytical quality-control (QC) evaluation [28].

Reference chemicals ametryn (P/N 45321) and 4-nitrotoluene (4NT; P/N N27322) were obtained from Sigma (St. Louis, MO, USA). Mass-labeled internal standards (ISs) obtained from Wellington Laboratories (Guelph, ON, Canada) were ^13^C_6–_4-nitrotoluene (Cambridge Isotope Labs, CLM-3913-S, Andover, MA, USA); Perfluoro-1-(^13^C_8_)octanesulfonamide (M8-FOSA-I); 2-Perfluorohexyl (1,1–^2^H_2_,1,2–^13^C_2_)ethanol (MFHET); 2-Perfluorobutyl (1,1,2,2–^2^H_4_) ethanol (MFBET); and 2-Perfluorooctyl (1,1–^2^H_2_,1,2–^13^C_2_)ethanol (MFOET).

### 2.2. PFAS Categorization

Using naming conventions proposed in earlier efforts [1,2], the selected PFAS were assigned to different categories as described in Table 1 and Table S1. Table 1 provides a high-level categorical listing, whereas Table S1 provides specific group assignments and additional structural information. Physicochemical descriptors were predicted using the open-source, OECD-compliant QSAR model OPEn structure-activity/property Relationship App (OPERA) [30,31], and van der Waals volumes were calculated in ChemAxon (ChemAxon, Budapest, Hungary). The number of carbons (C) present in each structure was identified.

### 2.3. Hepatocyte Clearance and Ultracentrifugation (UC) Assay Reagents

William’s Medium E (Gibco A1217601), dexamethasone, and cell maintenance cocktail B (Gibco CM4000) were obtained from Thermo Fisher Scientific (Waltham, MA, USA). Hepatosure OptiThaw media (P/N K8000; Lot No. 18–1–0322) was obtained from Sekisui Xenotech (Kansas City, KS, USA). Trypan blue solution (P/N 1450021) was obtained from Bio-Rad (Hercules, CA, USA). Pooled human cryopreserved primary hepatocyte suspensions were obtained from BioIVT (Westbury, NY, USA) and produced using nontransplantable tissue. The human 50-donor pool (mixed gender; Cat X0080005; Lot MYC), selected from BioIVT’s commercially available, prepooled lots, was selected following review of vendor-generated metabolic characterization information. Pooled, mixed-sex human plasma from de-identified donors was obtained from a commercial vendor that operates a U.S. Food and Drug Administration-licensed and inspected donor center (BioIVT bioivt.com (accessed 12 May 2023). Donors comprised five males and five females and ranged from 20 to 50 years of age. Plasma collected using anticoagulant K_2_EDTA was sterile-filtered (0.2 μM) and stored at <−70 °C until use.

### 2.4. UC Assay

The UC assay was developed based on earlier publications with some modification [32,33]. PFAS were grouped into sets of up to 5 chemicals based on chemical properties and analytical method constraints to obtain optimal analytical responses. Three replicates were run per experiment, with 4NT included as a reference compound in each to evaluate assay performance. All PFAS and 4NT, prepared at a 3 mM working stock, were added to human plasma to achieve a final, thoroughly mixed assay concentration of 10 μM, incubated at 37 °C for 1 h with shaking. After 1 h, one aliquot was collected (T1hr), a second aliquot continued to incubate at 37 °C, and the remainder underwent UC at 850,000× *g* for 4 h at 37 °C. After UC, the aqueous fraction (AF; i.e., supernatant devoid of plasma proteins/lipoproteins/fatty acids) was collected and transferred to a new tube. The second aliquot maintained at 37 °C was collected (T5hr). At the relevant time points, each volume of sample was combined with 3 volumes of vigorously mixed ice-cold acetonitrile with the labeled IS and stored for 10 min at −20 °C prior to centrifugation at 12,000× *g* for 10 min at 4 °C. The supernatant was collected and stored at <−70 °C until quantitative analysis. Fraction unbound in plasma (f_up_) is calculated from these samples by dividing the AF concentrations by the T5hr concentrations. Furthermore, chemical stability in plasma over the assay time course was assessed using the T1hr and T5hr samples.

To process samples for quantitative analysis, the frozen samples were thawed, mixed, and centrifuged at 12,000× *g* for 5 min (min). An aliquot of the supernatant was removed and diluted in acetonitrile (1:10) prior to instrument analysis.

### 2.5. Hepatic Metabolic Stability Assay

The hepatic metabolic stability assay measures the loss of a parent compound over time in a substrate-depletion approach [34]. Briefly, hepatocyte suspensions, after a viability assessment, were diluted in complete William’s medium E (containing dexamethasone and cell maintenance cocktail B) prior to addition to 96-well polypropylene plates containing an equal volume of media spiked with the test compound (1 μM assay concentration; assay cell density = 50,000 cells/100 μL) and maintained at 37 °C with shaking. At each time point, relevant plates were crashed with an equal volume of ice-cold acetonitrile, centrifuged, and supernatant transferred to a new plate prior to storage at <−70 °C until quantitative analysis. Controls, run in parallel, included cell-free (i.e., media only) and metabolically inactivated hepatocytes to evaluate abiotic chemical stability in the aqueous media matrix with and without cellular protein. In preliminary runs, these controls were run at T0 and T240 only. If significant instability was noted, the hepatocyte clearance assay was rerun, including cell-free samples at each time point and metabolically inactivated hepatocytes at selected time points. Ametryn was run concurrently as an assay reference compound to ensure hepatocytes were functioning and exhibiting clearance consistent with historical laboratory data.

To process samples for quantitative analysis, samples were thawed and mixed. An aliquot of sample was removed and diluted in acetonitrile (1 part sample plus 9 parts acetonitrile) containing the relevant ISs selected for the PFAS being analyzed (See Table S2). The target at instrument concentration of each IS was 10 pg/μL.

### 2.6. Quantitation using Gas Chromatography-Tandem Mass Spectrometry (GC-MS/MS) and GC(PCI)-MS

Sample analyses were performed on Agilent model 7890 and 8890 GC systems coupled with model 7010 triple quadrupole (QQQ) mass spectrometers. (Agilent Technologies, Santa Clara, CA, USA). Separation was performed using either Agilent DB-FFAP (30 m × 0.25 mm, 0.25 μm film, part No. 122–3232) or Agilent DB-624MS (30 m × 0.25 mm, 1.4 μm film, part No. 122–1334) fused silica GC columns with helium as the carrier gas at 1.2 mL/min. Regardless of method, samples were vortex-mixed at 3000 RPM for 10 s in both clockwise and counterclockwise directions prior to injection using the built-in vortex mixer on the Agilent 7693 autosampler trays fitted to each GC. Injection volume of 1 uL was injected onto an Agilent Ultra-Inert single-taper inlet liner containing deactivated glass wool (Part No. 5190–2293). Because multiple optimized methods were used depending on compound grouping, method-specific information is detailed in Table S2.

The Agilent 7010 GC/QQQ systems were operated in electron impact mode at 70 eV, using Agilent high-efficiency ion sources. The source temperature was 250 °C, with quadrupoles set to 150 °C. Instruments were tuned using high-sensitivity autotune prior to calibration, with gain set to 15 for all time segments. Acetonitrile solvent blanks and matrix blanks were analyzed throughout each run to evaluate any contamination or contribution from blanks to sample responses.

Similar analytical determinations utilizing GC(PCI)-MS were conducted on an Agilent 6890N GC interfaced with a 5975 MS, as described in [28]. Briefly, samples were introduced onto an RTX-1701 analytical column in pulsed splitless mode, in an inlet maintained at 200 °C. The initial oven temperature (38 °C) was held for 5 min before ramping at 5 °C/min to 90 °C, and then ramped to 250 °C at 10 °C/min with a hold of 3 min. All analyte concentrations were determined in selected-ion-monitoring (SIM) mode. The transfer line was maintained at 290 °C, and the source and quadrupoles were maintained at 250 and 150 °C, respectively.

Regardless of instrumentation used, samples were quantified using 15-point calibration curves from 1.75 to 1250 nM (in solution) for plasma protein binding, and from 7 to 5000 nM for hepatocyte clearance samples. The curves were generated using concentration/response ratios from the target analyte and IS, with either linear or quadratic regression models. The model and weighting, if necessary, were chosen to most accurately reflect the response of each compound and was evaluated by recalculating the individual calibration points using the curve. An acceptable model reflected an accuracy of ±30% of the theoretical value.

The estimated method detection limit (eMDL) for each analyte in each matrix was determined using both analytical platforms. This value is the minimum measured concentration of the chemical reported with 99% confidence to be distinguishable from a method blank (US EPA Method 821-R-16–006). To determine this value, a set of the lower-end calibration curve points were injected seven times. The eMDL is calculated by multiplying the standard deviation of their reported concentration by the one-tailed t-distribution test (for seven samples with six degrees of freedom: *t*-value of 3.14 at 99% confidence level). Determined MDLs guided identification of blank contamination or instrumental issues if presented. Additionally, using these calibration curve points, the estimated limit of quantitation (eLOQ) was determined as the concentration wherein all seven measures were within ±30% of the theoretical value.

### 2.7. Hepatic Metabolic Stability Assay Data Analysis

Hepatic metabolic clearance data were plotted in semi-log format (ln concentration vs. time) with three replicates at each time point, as previously described [35]. Linear regression analysis in conjunction with a standard F-test was used to determine whether the slope of the line (indicative of chemical clearance) was significantly different from 0. The equations described below are used to calculate the chemical half-life (T_½_) and intrinsic in vitro clearance (Cl_in vitro_) with units of μL/(min × million hepatocytes). In Equation (3), 2000 is a scalar used to adjust the assay cell number up to be consistent with units of 1 million cells in the Cl_int_ equation.

k = −(slope)(1)

(2) $$T_{\frac{1}{2}}=0.693/k$$

(3) $$Cl_{int}=\frac{\left((2000\right)\times 0.693)}{T_{\frac{1}{2}}}$$

Negative controls, including media only and metabolically inactivated hepatocyte controls, were included to monitor abiotic loss (i.e., background clearance) during the assay. Where significant compound loss occurred using the analysis described above, background clearance rates were calculated and used to background-adjust hepatic metabolic clearance rates. Where chemicals were cleared prior to the end of the analysis, at 240 min (T240), the corresponding negative control timepoint was used for background subtraction. Any chemicals displaying a 50% or more abiotic loss at T120 were deemed unstable and were excluded from further analyses.

### 2.8. Bayesian Modeling to Incorporate Experimental Uncertainty with Experimental Point Estimates

To estimate measurement uncertainty, a Bayesian analysis was performed on both sets of TK assay data using Markov chain Monte Carlo. The data were organized into a single file for each type of experimental analysis, provided as Supplementary Materials (Tables S6 and S7 for PPB and hepatic clearance data, respectively). The relationship between the parameters assumed to be involved in the measurement process was described as a graphic model in the JAGS language [36] interfaced through R [37]. For the PPB data, the basis of this model was the PPB Bayesian model [38], modified to reflect the UC assay and include calibration curves, as well as two statistical models being employed. The first statistical model describes the chemical-specific MS response factor (that is, the conversion factor between analyte peak ratio (to standard) and chemical concentration). The second model describes the relationship between the samples of the UC assay measurement. Both models were analyzed jointly with JAGS using five Markov chains. R package runjags [39] was used to repeatedly extend the Markov chains until the multivariate shrink factor calculated with all five chains was less than 1.05. Each time the chains were extended using a 50,000-iteration burn-in was followed by 50,000 iterations thinned to 2000 samples. In the event that measurements were made on multiple days, separate response calibrations were made, but a single f_up_ was estimated per chemical. The median and 95 percent credible interval (upper and lower bounds) were calculated from the final (converged) 10,000 samples from the five Markov chains. The analysis was performed using the EPA-developed R package invitroTKstats, which is available upon request.

### 2.9. In Vitro–In Vivo Extrapolation (IVIVE) and Administered Equivalent Dose (AED) Estimation

Using the experimentally generated PPB (fraction unbound in plasma, f_up_) and hepatic Cl_int_ data, IVIVE was performed as previously described [34] to calculate human steady-state plasma concentrations (C_ss_). Briefly, experimental measures of f_up_ are adjusted using blood:plasma partitioning information to estimate the fraction unbound in blood (f_ub_). Experimental hepatocyte Cl_int_ data are scaled up to represent whole-liver clearance (L/h). Assuming a 1 mg/kg/day dosage, C_ss_ values are estimated by incorporating hepatic clearance and nonmetabolic renal clearance (defined as glomerular filtration rate (GFR) multiplied by f_ub_), both adjusted for blood binding. Once estimated, these C_ss_ values are considered in conjunction with published in vitro bioactivity concentrations (i.e., lowest-observed effective concentrations, or LOECs) [40] to calculate AEDs (Table 2) [34]. See Table S10 for all relevant IVIVE inputs, scalars, calculations, and outputs.

### 2.10. Chemical Transformation Simulator Predictions

The chemical transformation simulator, a web-based, open-source tool to predict (bio)transformation products of chemicals following exposure to metabolic or environmental conditions [41] (https://qed.epa.gov/cts/; accessed on 14 September 2022), was used to identify potential products of PFAS following the loss of the parent analyte in either the aqueous abiotic assay media or the hepatocyte assay system. The PFAS reaction library [42] was employed within the reaction pathway simulator to derive the outputs, following either environmental or metabolic transformations. Outputs include transformation route, synthesis code, percent production, percent accumulation, and exact mass of products. Up to four generations were predicted.

### 2.11. Statistical Analyses

Nonlinear regression analyses conducted in GraphPad Prism v9.2.0 (GraphPad, San Diego, CA, USA) were employed in trends analyses.

## 3. Results

### 3.1. Analytical Method Development Outcomes

Of the 73 PFAS initially selected for TK data generation and quantitative method development, 13 were removed from the subsequent analysis due to their inability to achieve a reproducible, stable, and/or sufficiently sensitive response when diluted in acetonitrile and/or methanol in the initial method-development workflow employed for this effort. Most of these PFAS were alkanes or iodoalkanes that were predicted to have relatively low boiling points and/or high vapor pressures, which likely contributed to the challenges noted. For those analytes that yielded a reproducible and sensitive signal in the initial method-development phase, the next step was dilution in a relevant assay matrix to evaluate the impact of matrix effects on analyte sensitivity and stability. In the plasma mixed matrix utilized for the UC assay, 17 additional PFAS failed due to stability issues. Of these 17, some failed due to high instability—that is, rapid loss of the analyte signal due to degradation or loss at some point during solution preparation and/or sample processing—which prevented the development of a reliable quantitative method for any data generation. Others were sufficiently stable to allow for the analysis of UC assay samples, but in the end, the chemicals were not sufficiently stable during the assay to generate reliable f_up_ data. As a result, experimental f_up_ values were generated for 43 PFAS. During conduct of the hepatocyte clearance assay, 47 failed due to abiotic loss during the time course, as determined using negative no-cell controls containing only William’s E media. Hepatic Cl_int_ values were successfully measured for 13 PFAS. More details are provided in Tables S3 and S5.

### 3.2. Plasma Protein Binding Findings

**Figure 1:**
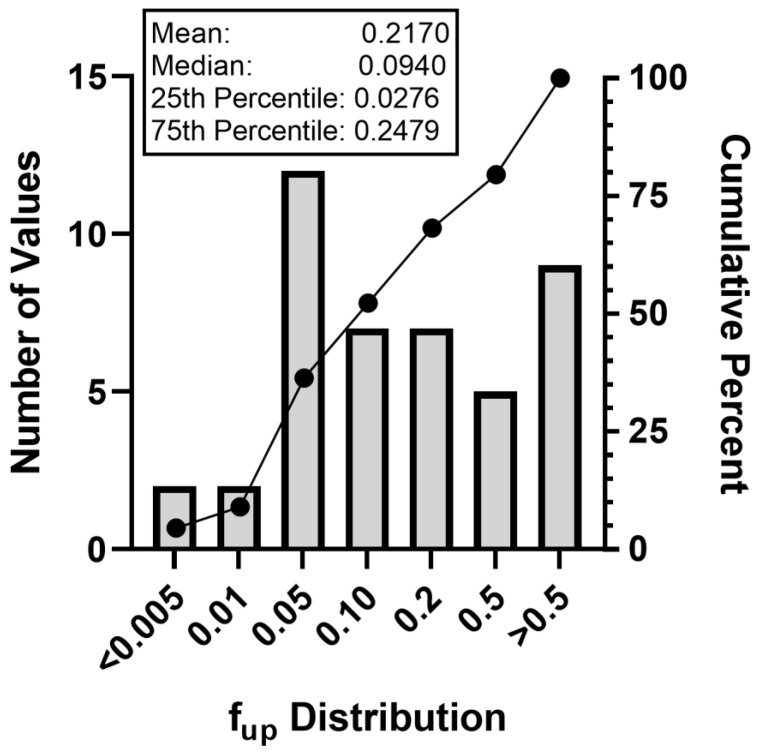
Plasma Protein Binding Distribution Across PFAS Test Set.

Experimental f_up_ values were derived for 43 PFAS. Values ranged from a minimum f_up_ of 0.004 for 1,6-dibromododecafluorohexane (DTXSID20335129) to 1 for 2-aminohexafluoropropan-2-ol (DTXSID80382093), with the 25th percentile and median values of 0.0276 and 0.0940, respectively (Figure 1). Comparing the median to an average f_up_ of 0.2170 demonstrates that the data were skewed toward being more highly bound, despite the range of binding noted across the larger dataset. Furthermore, comparing the median values for this set (f_up_ = 0.0940) with a second set of PFAS comprised primarily of perfluoroalkyl acids evaluated elsewhere (f_up_ = 0.005) [33], these PFAS exhibited on average a 20-fold lower binding.

### 3.3. Plasma Matrix Stability Observations

Percent chemical remaining at the end of the UC assay (T5hr/T1hr) was used to remove unstable chemicals from further evaluation. In the interest of retaining as much information as possible to inform TK and bioaccumulative potential, only PFAS exhibiting >60% loss were removed. Details are provided in Tables S3 and S4. Instability was noted for 17 PFAS: all except two displayed >99% loss of analyte at the T5hr point, with the remaining exhibiting 84 and 92% losses. Ten of the seventeen were PFAS acrylates, two contained ethanethiol functional groups, and the remainder had a mix of other functional groups (Table S3).

### 3.4. Impact of PFAS Chemical Space on Physicochemical Property–PPB Binding Trends and PPB Prediction Tool Performance

**Figure 2:**
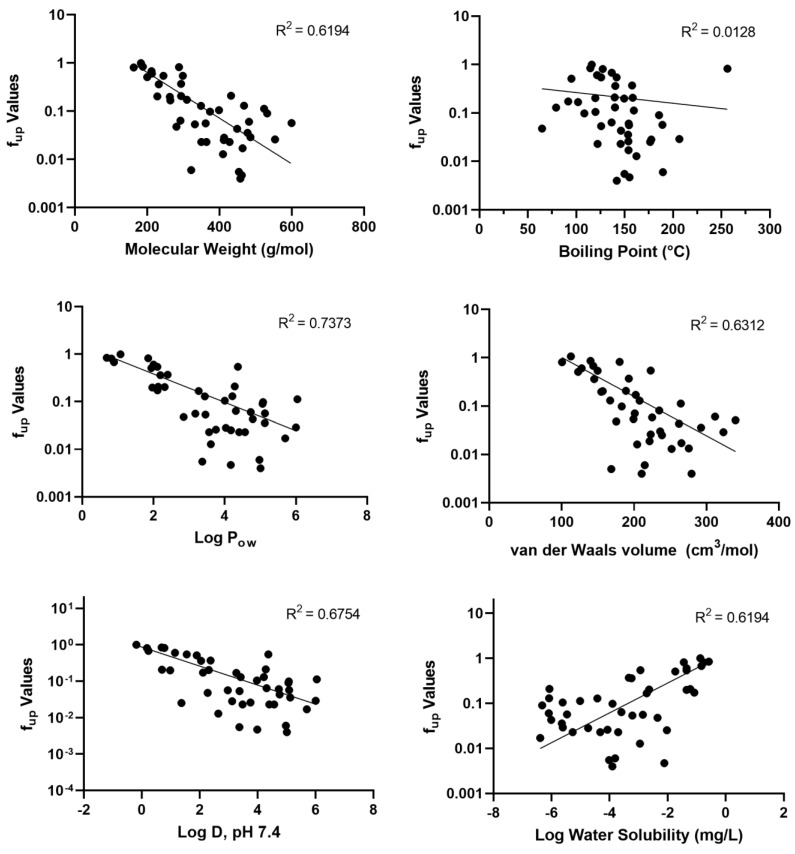
Trends Analyses of Physicochemical Properties and f_up_. Experimental fup and corresponding physicochemical property parameter are plotted for each PFAS (black dot). Nonlinear regression goodness of fit metrics (best-fit line, R^2^) are displayed for each analysis.

Six physicochemical properties and the experimental f_up_ data were evaluated using nonlinear regression for trends (Figure 2). Lower f_up_ values (i.e., higher binding) were reasonably correlated with increases in molecular weight (r^2^ = 0.6194), Log P_ow_ values (r^2^ = 0.7373), van der Waals volumes (r^2^ = 0.6312), and Log D at pH 7.4 (r^2^ = 0.6754). Alternately, higher f_up_ values were correlated with an increase in water solubility (r^2^ = 0.6194). No apparent trend emerged for boiling points.

**Figure 3:**
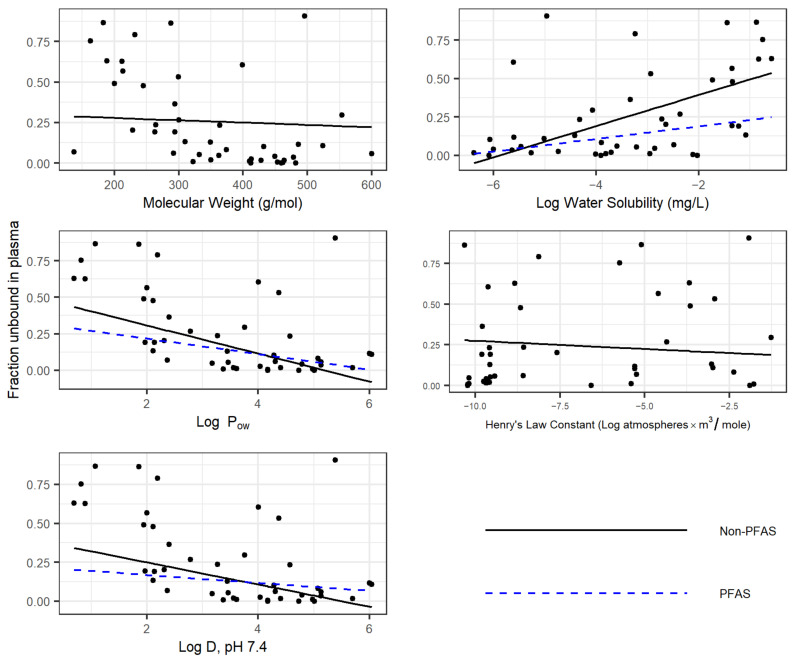
Trends Analyses of PFAS and non-PFAS Chemicals.

To evaluate whether PFAS physicochemical property PPB trends varied significantly from other chemicals, experimental f_up_ data were collected on (1) 1681 non-PFAS chemicals collected from the literature and present in R package “httk” [43]; (2) 114 PFAS (those evaluated here, in separate effort [33], and already present in R package “httk”; and (3) the 43 PFAS measured in this effort. For each set, the dependence of f_up_ on physicochemical properties was determined, and differences between the three sets were evaluated (Figure 3). For each property, a separate multivariate linear regression was performed for the measured f_up_ on the property and two set identifiers. The identifiers were yes/no factors indicating whether each compound was a PFAS and whether it was newly measured here. Statistical significance testing on the relationship between f_up_ and the property and any interactions with the set identifiers was performed using R function “lm”. For Log P_ow_, water solubility (LogWSol) and LogD74 (Log D at pH 7.4), a statistically significant different trend was observed for PFAS vs. non-PFAS chemicals, as evidenced by the blue line. No differences were observed for molecular weight or Henry’s Law constant. Moreover, in no cases were different trends observed between the 43 PFAS from this study and the 71 other PFAS.

The predictive performance of three quantitative structure property relationship (QSPR) tools designed to predict PPB (ADMET predictor (Simulations Plus); OPERA [31], and Dawson et al. [44]) were found to be weakly predictive of f_up_, with r^2^ values ranging from 0.2482 (OPERA v2.9) to 0.4853 (OPERA v2.6). ADMET predictor and the Dawson et al. [44] models had r^2^ values equal to 0.4104 and 0.4665, respectively.

### 3.5. Category-Based Evaluations of Plasma Protein Binding

**Figure 4:**
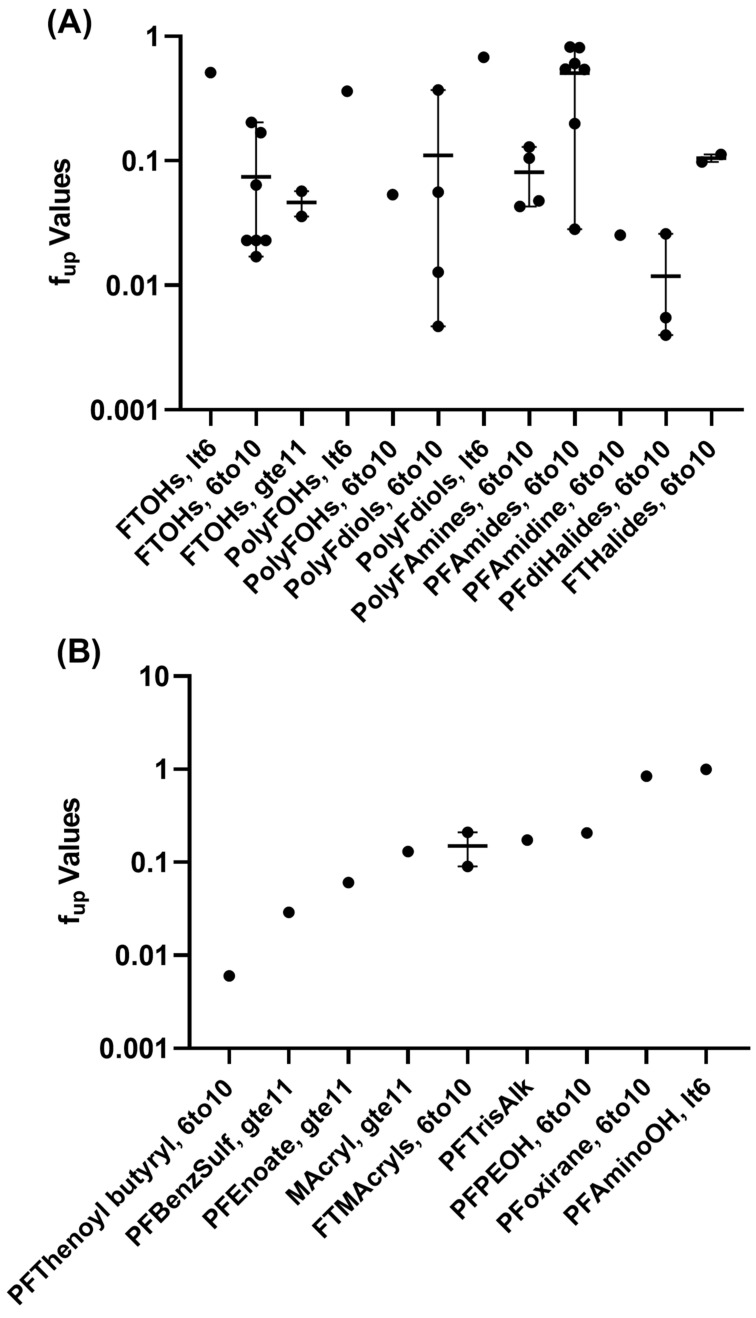
Plasma Protein Binding Evaluations by Functional Group. Experimental f_up_, mean (horizontal line), and range (vertical line) are displayed. (**A**) contains comparisons across PFAS containing alcohols, amines/amides, or halides (I, Br). (**B**) Excepting the fluorotelomer methacrylate group, B contains those with only one PFAS per group. Abbreviations are defined in Table S1.

Seventeen functional groups were represented across the forty-three PFAS successfully analyzed for PPB, with eight of the groups having a minimum of two structures per group. Groupings with the best coverage included the FT alcohols, polyfluorinated diols, perfluorinated amides, polyfluorinated amines, and perfluorodihalides (Figure 4). The perfluorodihalide group exhibited the highest binding, with a mean of f_up_ of 0.012 across the three structures analyzed. FT halides (specifically FT iodides) had a mean average that was 10-fold higher at 0.1. Perfluoroalkyl amides were the least bound, with a mean f_up_ = 0.51 (n = 7 structures). Polyfluorinated amines had an f_up_ = 0.0812 (n = 4).

### 3.6. Uncertainty Analysis

Uncertainty analysis was performed on the PPB and hepatic clearance experimental data (Tables S6–S9). Credible intervals found that five of the PPB estimates were uncertain—defined as a 95% credible interval ranging more than three orders of magnitude. Review of the experimental data revealed no clear trends regarding level of binding, with f_up_ values of the affected chemicals ranging from 0.005 to 0.3616 and a median f_up_ of 0.06. Experimental coefficients of variation (CVs) ranged from 2.63 to 26.42 (mean = 17.5). Follow-up analyses of hepatic clearance samples and abiotic loss monitoring confirmed the experimental findings, and Cl_int_ rates tracked with those derived using the traditional approach. Other outputs, along with metrics describing likelihood of degradation, can be found in Table S9.

### 3.7. Hepatic Metabolic Stability Findings and IVIVE Modeling

Hepatic metabolic stability was successfully quantitated for 13 PFAS. Six were polyfluorinated diols, five were perfluorinated amides, one was a fluorotelomer alcohol (4:2 fluorotelomer alcohol), and the last was 1H,1H,7H-perfluoroheptyl 4-methylbenzenesulfonate. Eleven of thirteen showed detectable clearance in the hepatocyte suspensions, including all but one of the amides and all alcohols (Table 2), with the highest Cl_int_ of 49.9 observed for 1H,1H,10H,10H-Perfluorodecane-1,10-diol. Using IVIVE, assuming an administered dosage of 1 mg/kg/day, C_ss_ values ranged from 0.19 to 2.69 μM for PFAS acids, and maxing out at 10.01 μM for 1H,1H,7H-perfluoroheptyl 4-methylbenzenesulfonate: the one PFAS predicted to be a neutral compound (Table 2 and Table S10). In vitro TK data obtained from earlier studies for PFAS legacy compounds, such as for PFOA (ammonium salt), PFOS, and PFOSA, were included for comparison [33,34]; these returned C_ss_ values in range or up to 68-fold higher: for instance 6.33 μM for PFOSA and 682 μM for PFOA, respectively.

Referencing in vitro bioactivity data generated using the BioSeek platform [40], the minimum lowest-observed effect concentration (LOEC) for each PFAS (ranging from 2 to 20 μM) was used to obtain the AEDs in Table 2. Resultant AEDs ranged from 0.70 (1H,1H,7H-perfluoroheptyl 4-methylbenzenesulfonate) to 101.04 mg/kg/day (heptafluorobutyramide). Comparisons to PFOA, for which the minimum LOEC was 2 μM and the AED was 0.003 mg/kg/day, demonstrate that the differences are driven primarily by chemical-specific TK.

### 3.8. Chemical Transformation Simulator (CTS) Findings

Compounds for which hepatic clearance was noted (Table 2) were run through the PFAS metabolism reaction library of CTS to predict likely metabolites. The amides were predicted to undergo amide hydrolysis to form their corresponding carboxylates: for instance, nonafluoropentanamide would form nonafluoropentanoic acid (Table S11). For 4:2 FTOH, four generations of metabolites were predicted, including formation of 4:2 fluorotelomer carboxylic acid, 4,4,5,5,6,6,6-heptafluoro-3-oxohexanoic acid, and several aldehyde intermediates. No CTS predictions were available for the PFAS diols, glycols, or 1H,1H,7H,7H-Perfluoroheptyl 4-methylbenzenesulfonate for which clearance was noted, indicating a lack of experimental literature data for these PFAS.

Reviewing the 18 PFAS that failed the experimental analysis due to instability, 13 were revealed to undergo hydrolysis. Ten of the thirteen were acrylates, wherein the present carboxylic acid ester was cleaved to form the respective carboxylic acid (Table S12). Others shown to be hydrolyzed included a propenoxide, epoxide, and an ethanol. The remaining four had no predicted reactions.

## 4. Discussion

PFAS provide unique and considerable challenges as a chemical family regulated under the Toxic Substances Control Act. As defined by the OECD, the 4730 structures span a wide range of chemical and functional spaces, from gaseous hydrofluorocarbons and perfluoroalkenes, liquid alcohols, to the highly persistent, solid PFAAs [10]. Across the exposures to the hazard continuum that are considered in risk evaluations, the routes of exposure, TK, and toxicity will require chemical or group-specific considerations to ensure adequate evaluation [27]. Unfortunately, little is known across the larger set of PFAS, with many PFAS protected as confidential business information, and others, designated as PFAA precursors, for which no research exists in the public domain [11,17]. Moreover, given the unique carbon:fluorine (C:F) backbone and amphiphilic nature, many fall well outside the chemical structures present in training sets used in quantitative structure property relationship (QSPR) development, leaving them outside the applicability domain of many in silico predictions [45,46]. For many PFAS, robust risk evaluations are not yet possible.

Plasma protein binding findings of this effort provide further confirmation that PFAS are high binders, with persistence being likely across the broader set. Comparing the median f_up_ of 0.094 (90.6% bound) to the average f_up_ of 0.2170 indicates that this set is skewed toward high binding, despite values ranging from 0.04 to 1 (Figure 1). Comparatively, these are 20-fold less-bound than 71 PFAS, comprised primarily of perfluoroalkyl acids and sulfonates, which exhibited a median f_up_ of 0.005 in a recent evaluation [33]. Despite these differences, once combined and compared to 1681 non-PFAS commercial chemicals (httk R package, v2.0; as evaluated in [34,35,38,47,48]), several aspects of PFAS physicochemical properties and TK are distinct, as evidenced by a median f_up_ of 0.13 versus a median of 0.0230 for the 114 PFAS and the significant differences in Log P_ow_, Log D, and water solubility (Figure 3). Furthermore, weak performances of available TK QSPRs to predict PFAS PPB underscores the value that these experimental data provide for accurate PFAS assessments. The retraining of such models will be an important next step.

Overall, 21 different functional categories were evaluated for plasma protein binding, with no strong, category-specific trends emerging (Figure 2). The perfluorodihalide group, defined as having a non-fluorine halide substitution group, exhibited the highest binding (mean f_up_ = 0.012). The lowest f_up_ (of 0.004) was observed for 1,6-dibromododecafluorohexane (DTXSID20335129). Fluorotelomer halides exhibited less binding on average (mean f_up_ = 0.11), which is consistent with the higher binding noted in other per- vs. polyfluoroalkyl substance comparisons [33]. Other than their use as an intermediate in chemical synthesis and as a precursor to the formation of other PFAS, little else is known about the commercial use or environmental fates of these PFAS [1,49]. Several PFAS alcohols were evaluated: two polyfluorinated diols exhibited high binding (f_up_ ≤ 0.01), but as a group, the binding for the four evaluated ranged from 0.005 to 0.6770. Six FTOHs, with a minimum of six carbons, exhibited varied binding (f_up_ 0.02–0.5). Increased detection of FTOHs in commercial products and in indoor air has heightened the need for more information on this set of PFAS [11,50].

Evaluation of the PFAS C_ss_ values derived using IVIVE provides useful lessons. In contrast to documented metabolic stability of legacy PFAS carboxylic acids [35,38], 11 of the 13 analyzed here showed significant clearance in hepatocytes. Assuming a 1 mg/kg/day administered dosage, IVIVE modeling estimates that the resulting parent PFAS C_ss_ values range from 0.19 to 10.01 μM, with a median of 0.43 μM (Table 2). 1H,1H,11H,11H-perfluorotetraethylene glycol, despite a relatively high clearance rate of 20 μL/min × million cells, was highly plasma-bound (f_up_ = 0.013), resulting in a C_ss_ of 1.50 μM. This is comparable to hexafluoroamylene glycol and pentafluoropropionamide (C_ss_ predicted at 2.1–2.7 μM), which exhibited no clearance but were relatively unbound (f_up_ = 0.68 or 0.81, respectively). Incorporating this information with in vitro potency measures from a recent in vitro PFAS screening effort by Houck et al. [40] demonstrates the significant role PFAS dosimetry plays in TK modeling: AEDs derived for GC-MS PFAS that were found to be equipotent to PFOA (2 μM LOEC, Table 2) had AEDs that were 327- to over 3500-fold higher than for PFOA (0.003 mg/kg/day). Thus, legacy PFAS carboxylates and sulfonates—for instance PFOA, PFOS (tested in Smeltz et al. [33])—are likely a higher priority for testing when parent compound dosimetry is considered.

The likelihood that PFAS will be metabolized to another more stable PFAS is an important consideration in any dosimetric evaluation. PFAS sulfonamides are in some instances used as precursor products, with their metabolism to carboxylic acids being an intended part of the manufacturing process. In this effort, hepatic metabolism was evaluated for five PFAS amides, with all but one demonstrating metabolism in hepatocyte suspensions. Although empirical metabolite identification data are lacking for these perfluoroalkyl amides, extensive studies of sulfonamides support the formation of respective carboxylic acids following amide hydrolysis in CTS [42]. Literature evaluations of perfluoroalkyl amides are limited to the medicinal chemistry space, where fluorination of bioactive compounds is a current trend to enhance compound stability and efficacy [51,52,53]. Furthermore, although several polyfluorinated alcohols demonstrated hepatic clearance (Table 2), beyond characterization of fluorotelomer alcohol metabolism [54], nothing is yet known regarding diol or glycol metabolite formation.

Whereas NAMs offer efficient tools to select high-priority chemicals for follow-up evaluation, consideration of assay amenability is paramount to ensure accurate interpretation of the resulting in vitro data. Physicochemical properties can broadly inform amenability, but, where feasible, a parallel analytical approach to monitor test agent levels in the assay system provides critical empirical information, particularly for emerging contaminants such as PFAS, in which their unique structures defy the described applicability domains of many QSAR/QSPR tools [45]. As the set evaluated here comprised the more volatile PFAS, abiotic loss was noted across many, underscoring the importance of time-matched negative or stability controls to avoid misinterpretation of data. Previous reports of in vitro data for 8:2 FTOH confirmed volatilization of the radiolabeled compound via headspace analysis; unfortunately, the same report mistakenly claimed that the respective carboxylic acid was not a significant transformation product [55]. In silico tools that predict PFAS bio(transformation) products, such as the chemical transformation simulator (Table S12) [42], provide useful information for identifying likely metabolites or hydrolysis products (Table S12).

This report provides in vitro plasma protein binding and hepatic clearance data for PFAS (43 and 13, respectively), for which minimal, if any, TK data had been previously reported. Adding data for 21 functional categories of PFAS, this information greatly expands the information for use in read-across grouping strategies and ongoing evaluations of PFAS hazards, dosimetry, and exposure. Leveraging new knowledge gained for these data-poor PFAS presents valuable insight as scientists seek to identify and prioritize PFAS groupings for robust exposure characterization, environmental fates and degradation studies, biomonitoring studies, and/or toxicity testing.

## Figures and Tables

**Table 1:** PFAS Analyzed in this Effort.

PFAS Groupings with Uses in Consumer and Manufacturing Products			
PFAS Group	Chemicals Present in Study	Example(s)	Uses *
Fluorotelomer Alcohols (FTOHs)	10	4:2 FTOH; 6:2 FTOH; 8:2 FTOH	Coatings, waxes, paints, varnishes, and inks (nonstick cookware); cosmetic and personal care products (foundation, mascara); fire-fighting foams; medical uses (dental care products); paper and cardboard packaging; plastics, resins, and rubber; sporting goods; and textiles (antifogging cloths, apparel).
Other Polyfluorinated Alcohols	10	Dodecafluoroheptanol; Perfluoropinacol	Coatings, waxes, paints, varnishes, and inks; cosmetic and personal care products (mascara); oil and gas surfactants; paper and cardboard packaging; photography agents; plastics, resins, and rubber; semiconductor surfactants; textiles; and transportation materials.
Acrylates (methacrylates, diacrylates)	12	8:2 Fluorotelomer acrylate; 6:2 Fluorotelomer methacrylate	Coatings, waxes, paints, varnishes, and inks; cosmetic and personal care products (hair care items); fire-fighting foams; medical uses (contact lenses); paper and cardboard packaging; plastics, resins, and rubber; textiles (apparel); and transportation materials.
Halides (Br, I)	9	Perfluoro-1,4-diiodobutane; 1,6-Dibromododecafluorohexane	Coatings, waxes, paints, varnishes, and inks; oil and gas surfactants; paper and cardboard packaging; pesticide and fertilizer formulations; photography agents (contrast agents); plastics, resins, and rubber; refrigerants; semiconductor surfactants; textiles; and transportation materials.
Amines	6	1H,1H-Perfluoroheptylamine	Dry-cleaning systems; electronics (fluids); fire-fighting foams; medical uses (oxygen carriers in cells, pharmaceutical processing aids); oil and gas surfactants; paper and cardboard packaging; photography agents; semiconductor surfactants; and textiles.
Amides	7	Heptafluorobutyramide	Coatings, waxes, paints, varnishes, and inks; cosmetic and personal care products (hair care products); fire-fighting foams; oil and gas surfactants; paper and cardboard packaging; pesticide and fertilizer formulations; photography agents; semiconductor surfactants; textiles (apparel); and transportation materials.
Alkanes	2	1-(Perfluorohexyl)octane	Coatings, waxes, paints, varnishes, and inks; cosmetic and personal care products (make-up, creams); medical uses (ophthalmological surgical aid); refrigerants; scientific materials (tracing agents); and sporting goods (ski wax).
Ethers, Esters, and Ethoxylates	10	Methyl 2H,2H,3H,3H-perfluoroheptanoate; tris(Trifluoroethoxy)methane; 1H,1H-Heptafluorobutyl epoxide	Coatings, waxes, paints, varnishes, and inks; cosmetic and personal care products (soap, shampoo); fire-fighting foams; medical uses (contact lenses); mining; oil and gas surfactants; paper and cardboard packaging; photography agents; plastics, resins, and rubber; semiconductor surfactants; textiles; and transportation materials.
Silanes	2	Trichloro((perfluorohexyl)ethyl) silane	Coatings, waxes, paints, varnishes, and inks; cosmetic and personal care products (make-up, moisturizers, lip balm); semiconductor surfactants; and textiles.
Sulfur-containing	4	2-(Perfluorooctyl)ethanthiol	Cosmetic and personal care products; fire-fighting foams; paper and cardboard packaging; textiles; and transportation materials.
Diketones	1	(Heptafluorobutanoyl)pivaloyl methane	Fire-fighting foams and textiles.

* This is not a complete list of every use for all PFAS that could be characterized with these groupings, but rather an illustration of the variety of potential sources and exposures to the PFAS examined in this study as described in recent analyses [11,12,13,14,15,16,17].

**Table 2:** In Vitro–In Vivo Extrapolation to Estimate C_ss_ and Comparisons to HTS Bioactivity.

DTXSID	Compound Name	Mol. Wt. (g/mol)	f_up_	In Vitro Cl_int_ (μL/min/10^6^ cells) ^a^	Cl_renal_ (L/h) ^a^	Cl_hep_ (L/h) ^a^	C_ss_ (μM) ^a^	LOEC (μM) ^b^	AED (mg/kg/Day)
DTXSID3059927	Hexafluoroamylene glycol	212.09	0.6770	0.00	6.7	0.000	2.07	2	0.966
DTXSID0059871	Pentafluoropropionamide	163.05	0.8089	0.00	6.7	0.000	2.69	20	7.431
DTXSID80310730	Octafluoroadipamide	288.10	0.8201	2.95	6.7	23.099	0.34	2	5.840
DTXSID10382147	3-(Perfluoro-2-butyl) propane-1,2-diol	294.12	0.3696	5.88	4.5024	28.460	0.30	2	6.595
DTXSID70381090	1H,1H,8H,8H-Perfluoro-3,6-dioxaoctane-1,8-diol	294.10	0.2069	6.72	2.5204	20.549	0.43	7	16.154
DTXSID70366226	Perfluoropentanamide	245.07	0.5417	14.68	6.5989	56.570	0.19	7	36.860
DTXSID00380798	1H,1H,11H,11H-Perfluorotetraethylene glycol	410.11	0.0128	19.93	0.1559	4.634	1.50	2	1.336
DTXSID2060965	Heptafluorobutyramide	213.06	0.6030	19.95	6.7	63.013	0.20	20	101.039
DTXSID30396867	1H,1H,8H,8H-Perfluorooctane-1,8-diol	362.12	0.0560	20.01	0.6822	17.326	0.45	7	15.526
DTXSID30340244	1H,1H,7H-Perfluoroheptyl 4-methylbenzenesulfonate	486.27	0.0290	23.85	0.0158	0.588	10.01	7	0.700
DTXSID60400587	Nonafluoropentanamide	263.03	0.1985	26.03	2.4181	47.130	0.23	7	31.034
DTXSID1062122	4:2 Fluorotelomer alcohol	264.09	0.1680	37.71	2.0465	51.672	0.21	2	9.651
DTXSID50369896	1H,1H,10H,10H-Perfluorodecane-1,10-diol	462.13	0.0047	49.86	0.0573	4.275	1.47	2	1.362
DTXSID8037708	Ammonium perfluorooctanoate (PFOA) ^c^	414.07	0.0006	0.27	0.0073	0.003	681.96	2	0.003
DTXSID8037706	Potassium perfluorooctanesulfonate (PFOS) ^c^	500.13	0.0040	1.81	0.0487	0.138	31.41	7	0.223
DTXSID3038939	Perfluorooctanesulfonamide ^c^	499.15	0.0039	11.93	0.0475	0.882	6.33	2	0.316

^a^ See Materials and Methods for details and Table S10 for scalars, inputs, and equations. ^b^ Values from [40]. ^c^ f_up_ from [33] and Cl_int_ from [34].

## Data Availability

Given the funding of this effort by the US EPA and in compliance with the US EPA public access policy, the accepted, nonformatted version of the accepted manuscript and any associated data files will be made available on PubMed Central one year after acceptance by the journal.

## Supplementary Materials

The following supporting information can be downloaded at: https://www.mdpi.com/article/10.3390/toxics11050463/s1, Table S1. PFAS Info–Identifiers, Category Groupings, Physchem Properties. Table S2. GC-MS/MS Instrument Parameters. Table S3. TK Data Summary. Table S4. UC Experimental Data. Table S5. Hep Clint Experimental Data. Table S6. UC Level 2 Bayes Inputs. Table S7. Hep Cint Level 2 Bayes Inputs. Table S8. UC Level 4 Bayes Outputs. Table S9. Clint Level 4 Bayes Outputs. Table S10. IVIVE Calculations. Table S11. CTS Metab Outputs. Table S12. Fst_CTS_Env_Outputs.
